# 6-Bromo-1*H*-indole-3-carb­oxy­lic acid

**DOI:** 10.1107/S1600536812006381

**Published:** 2012-03-10

**Authors:** Jing Zhao, Yan Wang

**Affiliations:** a Ordered Matter Science Research Center, College of Chemistry and Chemical Engineering, Southeast University, Nanjing 211189, People’s Republic of China

## Abstract

In the title mol­ecule, C_9_H_6_BrNO_2_, the dihedral angle between the –COOH group and the ring system is 6 (4)°. In the crystal, pairs of O—H⋯O hydrogen bonds link the mol­ecules into inversion dimers and these dimers are connected *via* N—H⋯O hydrogen bonds to form layers parallel to the (-101) plane.

## Related literature

For related literature, see: Lang *et al.* (2011 ▶); Luo *et al.* (2011 ▶).
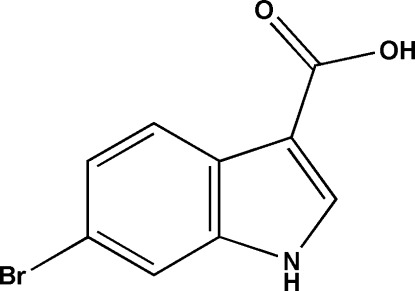


## Experimental

### 

#### Crystal data

C_9_H_6_BrNO_2_
*M* *_r_* = 240.06
Monoclinic, 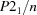

*a* = 7.2229 (14) Å
*b* = 11.874 (2) Å
*c* = 11.079 (2) Å
β = 108.37 (3)°
*V* = 901.7 (3) Å^3^
*Z* = 4
Mo *K*α radiation
μ = 4.52 mm^−1^
*T* = 293 K
0.30 × 0.23 × 0.20 mm

#### Data collection

Rigaku SCXmini diffractometer
Absorption correction: multi-scan (*CrystalClear*; Rigaku, 2005 ▶) *T*_min_ = 0.977, *T*_max_ = 0.984
8876 measured reflections
2051 independent reflections
1284 reflections with *I* > 2σ(*I*)
*R*_int_ = 0.082

#### Refinement

*R*[*F* ^2^ > 2σ(*F* ^2^)] = 0.063
*wR*(*F* ^2^) = 0.158
*S* = 1.06
2051 reflections
122 parameters
H atoms treated by a mixture of independent and constrained refinement
Δρ_max_ = 0.52 e Å^−3^
Δρ_min_ = −0.76 e Å^−3^

### 

Data collection: *CrystalClear* (Rigaku, 2005 ▶); cell refinement: *CrystalClear*; data reduction: *CrystalClear*; program(s) used to solve structure: *SHELXS97* (Sheldrick, 2008 ▶); program(s) used to refine structure: *SHELXL97* (Sheldrick, 2008 ▶); molecular graphics: *DIAMOND* (Brandenburg & Putz, 2005 ▶); software used to prepare material for publication: *SHELXL97*.

## Supplementary Material

Crystal structure: contains datablock(s) I, global. DOI: 10.1107/S1600536812006381/aa2041sup1.cif

Structure factors: contains datablock(s) I. DOI: 10.1107/S1600536812006381/aa2041Isup2.hkl

Supplementary material file. DOI: 10.1107/S1600536812006381/aa2041Isup3.cml

Additional supplementary materials:  crystallographic information; 3D view; checkCIF report

## Figures and Tables

**Table 1:** Hydrogen-bond geometry (Å, °)

*D*—H⋯*A*	*D*—H	H⋯*A*	*D*⋯*A*	*D*—H⋯*A*
O2—H7⋯O1^i^	0.97 (9)	1.67 (10)	2.627 (5)	169 (8)
N1—H1*A*⋯O1^ii^	0.86	2.16	2.928 (6)	148

Symmetry codes: (i) 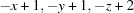; (ii) 
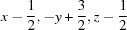
.

**Fig. 1.**
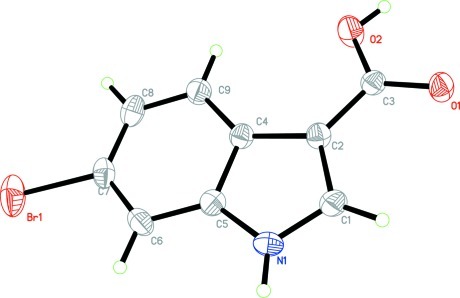
The molecular structure of the title compound with the atom labelling scheme. Displacement ellipsoids are drawn at 30% probability level.

**Fig. 2.**
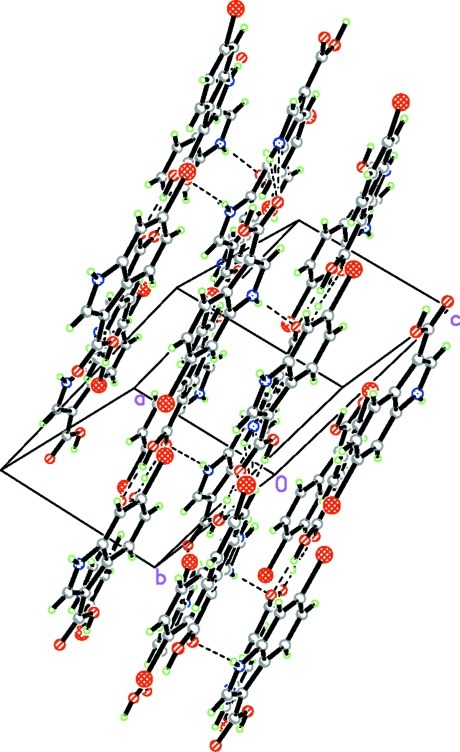
A packing diagram of the title compound. Intermolecular hydrogen bonds are shown as dashed lines.

## supplementary crystallographic information

### Comment

Indole derivatives such as indole-3-carboxylates are important building blocks in the synthesis of many pharmaceuticals and biologically active compounds. (Lang *et al.*, 2011; Luo,*et al.*, 2011). In the crystal structure of the title compound (Fig. 1), intermolecular O—H···O hydrogen bonds link the molecules into dimers and the dimers are connected *via* intermolecular N—H···O hydrogen bonds forming layers parallel to (101) plane (Table 1, Fig. 2).

### Experimental

A solution of the title compound (0.2 g) in methanol (20 ml) was placed in a dark place. Yellow single crystals suitable for X-ray diffraction study were obtained by slow evaporation of the solution over a period of 7 d.

### Refinement

H atoms attached to C and N were placed into calculated positions and treated as riding with C—H = 0.93 Å, N—H = 0.86 Å and *U*_iso_(H) = 1.2*U*_eq_(C, N). Carboxylic H atom was found from difference maps and refined independently.

### Crystal data

**Table:**

C_9_H_6_BrNO_2_	*F*(000) = 472
*M**_r_* = 240.06	*D*_x_ = 1.768 Mg m^−^^3^
Monoclinic, *P*2_1_/*n*	Mo *K*α radiation, λ = 0.71073 Å
Hall symbol: -P 2yn	Cell parameters from 2051 reflections
*a* = 7.2229 (14) Å	θ = 3.0–27.5°
*b* = 11.874 (2) Å	µ = 4.52 mm^−^^1^
*c* = 11.079 (2) Å	*T* = 293 K
β = 108.37 (3)°	Prism, brown
*V* = 901.7 (3) Å^3^	0.30 × 0.23 × 0.20 mm
*Z* = 4	

### Data collection

**Table:**

Rigaku SCXmini diffractometer	2051 independent reflections
Radiation source: fine-focus sealed tube	1284 reflections with *I* > 2σ(*I*)
Graphite monochromator	*R*_int_ = 0.082
Detector resolution: 13.6612 pixels mm^-1^	θ_max_ = 27.5°, θ_min_ = 3.0°
CCD_Profile_fitting scans	*h* = −9→9
Absorption correction: multi-scan (*CrystalClear*; Rigaku, 2005)	*k* = −15→14
*T*_min_ = 0.977, *T*_max_ = 0.984	*l* = −14→14
8876 measured reflections	

### Refinement

**Table:**

Refinement on *F*^2^	Primary atom site location: structure-invariant direct methods
Least-squares matrix: full	Secondary atom site location: difference Fourier map
*R*[*F*^2^ > 2σ(*F*^2^)] = 0.063	Hydrogen site location: inferred from neighbouring sites
*wR*(*F*^2^) = 0.158	H atoms treated by a mixture of independent and constrained refinement
*S* = 1.06	*w* = 1/[σ^2^(*F*_o_^2^) + (0.0513*P*)^2^ + 1.7606*P*] where *P* = (*F*_o_^2^ + 2*F*_c_^2^)/3
2051 reflections	(Δ/σ)_max_ < 0.001
122 parameters	Δρ_max_ = 0.52 e Å^−^^3^
0 restraints	Δρ_min_ = −0.76 e Å^−^^3^

### Special details

**Table:**

Geometry. All e.s.d.'s (except the e.s.d. in the dihedral angle between two l.s. planes) are estimated using the full covariance matrix. The cell e.s.d.'s are taken into account individually in the estimation of e.s.d.'s in distances, angles and torsion angles; correlations between e.s.d.'s in cell parameters are only used when they are defined by crystal symmetry. An approximate (isotropic) treatment of cell e.s.d.'s is used for estimating e.s.d.'s involving l.s. planes.
Refinement. Refinement of *F*^2^ against ALL reflections. The weighted *R*-factor *wR* and goodness of fit *S* are based on *F*^2^, conventional *R*-factors *R* are based on *F*, with *F* set to zero for negative *F*^2^. The threshold expression of *F*^2^ > σ(*F*^2^) is used only for calculating *R*-factors(gt) *etc*. and is not relevant to the choice of reflections for refinement. *R*-factors based on *F*^2^ are statistically about twice as large as those based on *F*, and *R*-factors based on ALL data will be even larger.

### Fractional atomic coordinates and isotropic or equivalent isotropic displacement parameters (Å^2^)

**Table:**

	*x*	*y*	*z*	*U*_iso_*/*U*_eq_	
Br1	0.08018 (10)	0.38905 (7)	0.15536 (6)	0.0777 (4)	
C5	0.2174 (7)	0.5896 (4)	0.4756 (5)	0.0394 (12)	
O2	0.4390 (7)	0.4389 (3)	0.8444 (4)	0.0571 (12)	
O1	0.4564 (6)	0.6167 (3)	0.9140 (3)	0.0453 (9)	
C3	0.4157 (7)	0.5477 (4)	0.8231 (5)	0.0369 (12)	
N1	0.2185 (7)	0.6961 (4)	0.5252 (4)	0.0457 (11)	
H1A	0.1800	0.7563	0.4813	0.055*	
C4	0.2939 (7)	0.5144 (4)	0.5765 (5)	0.0366 (11)	
C9	0.3036 (8)	0.4004 (5)	0.5495 (5)	0.0452 (13)	
H9A	0.3525	0.3484	0.6146	0.054*	
C8	0.2397 (9)	0.3663 (5)	0.4251 (6)	0.0504 (14)	
H8A	0.2447	0.2902	0.4063	0.060*	
C2	0.3405 (7)	0.5824 (4)	0.6922 (5)	0.0361 (11)	
C1	0.2896 (7)	0.6912 (4)	0.6539 (5)	0.0421 (13)	
H1B	0.3021	0.7525	0.7082	0.051*	
C7	0.1676 (7)	0.4426 (5)	0.3265 (5)	0.0440 (13)	
C6	0.1550 (7)	0.5551 (5)	0.3481 (5)	0.0459 (14)	
H6A	0.1075	0.6061	0.2818	0.055*	
H7	0.484 (13)	0.427 (7)	0.936 (9)	0.13 (3)*	

### Atomic displacement parameters (Å^2^)

**Table:**

	*U*^11^	*U*^22^	*U*^33^	*U*^12^	*U*^13^	*U*^23^
Br1	0.0665 (5)	0.1168 (7)	0.0510 (4)	−0.0166 (4)	0.0201 (3)	−0.0338 (4)
C5	0.037 (3)	0.040 (3)	0.041 (3)	0.000 (2)	0.012 (2)	−0.002 (2)
O2	0.095 (3)	0.029 (2)	0.041 (2)	−0.002 (2)	0.012 (2)	0.0027 (17)
O1	0.065 (2)	0.0304 (19)	0.038 (2)	−0.0045 (18)	0.0119 (18)	−0.0017 (15)
C3	0.039 (3)	0.032 (3)	0.039 (3)	0.000 (2)	0.011 (2)	−0.001 (2)
N1	0.057 (3)	0.035 (2)	0.043 (3)	0.009 (2)	0.014 (2)	0.011 (2)
C4	0.038 (3)	0.035 (3)	0.037 (3)	−0.001 (2)	0.013 (2)	−0.001 (2)
C9	0.055 (3)	0.041 (3)	0.042 (3)	0.003 (3)	0.018 (3)	0.003 (2)
C8	0.058 (4)	0.044 (3)	0.055 (4)	−0.008 (3)	0.026 (3)	−0.015 (3)
C2	0.039 (3)	0.032 (3)	0.037 (3)	−0.004 (2)	0.012 (2)	−0.001 (2)
C1	0.047 (3)	0.032 (3)	0.044 (3)	0.001 (2)	0.011 (3)	−0.002 (2)
C7	0.039 (3)	0.060 (4)	0.036 (3)	−0.009 (3)	0.016 (2)	−0.010 (3)
C6	0.040 (3)	0.064 (4)	0.031 (3)	0.003 (3)	0.008 (2)	0.006 (3)

### Geometric parameters (Å, º)

**Table:**

Br1—C7	1.908 (5)	C4—C9	1.394 (7)
C5—N1	1.377 (7)	C4—C2	1.461 (7)
C5—C6	1.402 (7)	C9—C8	1.369 (8)
C5—C4	1.401 (7)	C9—H9A	0.9300
O2—C3	1.315 (6)	C8—C7	1.388 (8)
O2—H7	0.97 (9)	C8—H8A	0.9300
O1—C3	1.259 (6)	C2—C1	1.374 (7)
C3—C2	1.439 (7)	C1—H1B	0.9300
N1—C1	1.356 (6)	C7—C6	1.365 (8)
N1—H1A	0.8600	C6—H6A	0.9300
			
N1—C5—C6	129.0 (5)	C9—C8—C7	121.5 (5)
N1—C5—C4	108.4 (4)	C9—C8—H8A	119.2
C6—C5—C4	122.6 (5)	C7—C8—H8A	119.2
C3—O2—H7	108 (5)	C1—C2—C3	124.0 (5)
O1—C3—O2	120.8 (5)	C1—C2—C4	106.5 (4)
O1—C3—C2	122.6 (4)	C3—C2—C4	129.4 (5)
O2—C3—C2	116.6 (5)	N1—C1—C2	109.9 (5)
C1—N1—C5	109.5 (4)	N1—C1—H1B	125.0
C1—N1—H1A	125.3	C2—C1—H1B	125.0
C5—N1—H1A	125.3	C6—C7—C8	122.0 (5)
C9—C4—C5	118.8 (5)	C6—C7—Br1	118.7 (4)
C9—C4—C2	135.4 (5)	C8—C7—Br1	119.3 (4)
C5—C4—C2	105.8 (4)	C7—C6—C5	116.4 (5)
C8—C9—C4	118.7 (5)	C7—C6—H6A	121.8
C8—C9—H9A	120.7	C5—C6—H6A	121.8
C4—C9—H9A	120.7		

### Hydrogen-bond geometry (Å, º)

**Table:**

*D*—H···*A*	*D*—H	H···*A*	*D*···*A*	*D*—H···*A*
O2—H7···O1^i^	0.97 (9)	1.67 (10)	2.627 (5)	169 (8)
N1—H1*A*···O1^ii^	0.86	2.16	2.928 (6)	148

Symmetry codes: (i) −*x*+1, −*y*+1, −*z*+2; (ii) *x*−1/2, −*y*+3/2, *z*−1/2.
